# A RAD Tag Derived Marker Based Eggplant Linkage Map and the Location of QTLs Determining Anthocyanin Pigmentation

**DOI:** 10.1371/journal.pone.0043740

**Published:** 2012-08-17

**Authors:** Lorenzo Barchi, Sergio Lanteri, Ezio Portis, Giampiero Valè, Andrea Volante, Laura Pulcini, Tommaso Ciriaci, Nazareno Acciarri, Valeria Barbierato, Laura Toppino, Giuseppe Leonardo Rotino

**Affiliations:** 1 DIVAPRA Plant Genetics and Breeding, University of Torino, Grugliasco, Torino, Italy; 2 CRA-GPG Genomic Research Centre, Fiorenzuola d'Arda, Piacenza, Italy; 3 CRA-RIS, Rice Research Unit, Vercelli, Italy; 4 CRA-ORA Research Unit for Vegetable Crops, Monsampolo del Tronto, Ascoli Piceno, Italy; 5 CRA-ORL Research Unit for Vegetable Crops, Montanaso Lombardo, Lodi, Italy; Max Planck Institute for Chemical Ecology, Germany

## Abstract

Both inter- and intra-specific maps have been developed in eggplant (*Solanum melongena* L.). The former benefit from an enhanced frequency of marker polymorphism, but their relevance to marker-assisted crop breeding is limited. Combining the restriction-site associated DNA strategy with high throughput sequencing has facilitated the discovery of a large number of functional single nucleotide polymorphism (SNP) markers discriminating between the two eggplant mapping population parental lines ‘305E40’ and ‘67/3’. A set of 347 *de novo* SNPs, together with 84 anchoring markers, were applied to the F_2_ mapping population bred from the cross ‘305E40’ x ‘67/3’ to construct a linkage map. In all, 415 of the 431 markers were assembled into twelve major and one minor linkage group, spanning 1,390 cM, and the inclusion of established markers allowed each linkage group to be assigned to one of the 12 eggplant chromosomes. The map was then used to discover the genetic basis of seven traits associated with anthocyanin content. Each of the traits proved to be controlled by between one and six quantitative trait loci (QTL), of which at least one was a major QTL. Exploitation of syntenic relationships between the eggplant and tomato genomes facilitated the identification of potential candidate genes for the eggplant QTLs related to anthocyanin accumulation. The intra-specific linkage map should have utility for elucidating the genetic basis of other phenotypic traits in eggplant.

## Introduction

The eggplant (*Solanum melongena* L.) is the third most important solanaceous crop (after potato and tomato, see http://faostat.fao.org), with a global production level in 2010 of ∼41.8Mt. It is an important vegetable in south Asia, the Middle and Near East, Africa and Southern Europe [1]. Its fruit shape varies from round to elongated, and is a good source of dietary minerals and vitamins. Despite the economic and nutritional importance of eggplant, its genome organization is much less well explored than that of the other solanaceous crops tomato and potato, for which a complete genomic sequence is now available (http://solgenomics.net/genomes). The eggplant is an autogamous diploid (2*n* = 2*x*  = 24), with a haploid DNA content of 1.2pg [2], equivalent to a genome size of 1.1Gbp, analogous to the one of tomato (∼950 Mbp) and potato (∼850 Mbp), and about one third of the genome of sweet pepper. The earliest linkage map constructed for eggplant was based on the RFLP genotyping of a small F_2_ population bred from the interspecific cross *S. linneanum* x *S. melongena* [3]; this map has since been updated by Wu et al. [4] by the addition of a set of conserved orthologous markers. The earliest purely intraspecific map was based on various PCR-based markers [5], [6], and was later supplemented by a large number of additional microsatellite loci to give an overall map length of 959 cM [7]. The most recently published intraspecific map (1,285 cM) was developed from two F_2_ populations using a mixture of microsatellites and gene-based markers, mostly derived from putative orthologs among eggplant, tomato and potato [8]. The latter map has been exploited to map quantitative trait loci (QTL) associated with parthenocarpy [9].

The Barchi et al. [10] intraspecific F_2_ mapping population is based on 238 markers, and spans 719 cM. Its value lies in its parental lines having been subjected to a large-scale single nucleotide polymorphism (SNP) discovery exercise, achieved by combining the “Restriction-site Associated DNA” (RAD, [11]) method with high throughput Illumina DNA sequencing [12]. The resulting sequence dataset consists of ∼45,000 non-redundant sequences, of which ∼29% are putative coding sequences; ∼30% of the sequences are informative between the parental pair, yielding a resource of ∼10,000 SNPs, almost nearly 1,000 indels and 1,800 putative microsatellites. The high throughput sequencing of the RAD tags enabled the development of useful markers for extending the current knowledge of the genome organization of eggplant and for carrying out comparative genomic analyses within the *Solanaceae* family.

Here we report the construction of a new intraspecific eggplant linkage map, mostly based on RAD tag derived SNP markers. The resulting map was used to identify a number of QTLs underlying anthocyanin pigmentation.

## Methods

### Permission

No specific permits were required for the described field studies, which took place in two experimental fields at the CRA-ORL in Montanaso Lombardo and CRA-ORA in Monsampolo del Tronto. These field plots were used by the authors of this paper affiliated to the aforementioned institution (LP, TC, NA, VB, LT and GLR) for field trials for phenotypic characterization of eggplant mapping populations.

### Plant materials and DNA isolation

A population of 156 F_2_ plants was bred from the cross ‘305E40’ x ‘67/3’, breeding lines which differ from one another with respect to a number of key agronomic traits. The highly homozygous female parent (‘305E40’) forms a pink corolla and produces long, highly pigmented dark purple fruit. The line was derived from a somatic hybrid between the cultivar ‘Dourga’ and *S. aethiopicum.* Its pedigree also includes cvs. ‘DR2’ and ‘Tal1/1’ [13], [14]. ‘67/3’ produces more anthocyanin than ‘305E40’ in its leaves and stems, its corolla colour is violet, and it produces round, violet coloured fruit. The line is an F_8_ selection from the intra-specific cross cv. ‘Purpura’ x cv. ‘CIN2’. DNA samples were extracted from young leaves, using the GenElute^TM^ Plant Genomic DNA Miniprep kit (Sigma, St. Louis, MO), following the manufacturer's protocol.

### Marker data generation, map construction and BLAST search

The mapping population was genotyped with respect to 472 markers, comprising 388 SNPs, 43 microsatellites, three CAPS,11 RFLPs and 27 COSII markers. Of the SNPs, 384 were those selected by Barchi et al. [12] from RAD tag derived sequence. Genotyping was achieved using the GoldenGate assay (Illumina, San Diego, CA), UC Davis Genome Center with automatic allele calling implemented with GenCall software (Illumina). Two of the F2 progeny were represented twice in each genotyping assay to provide an internal control. The remaining four SNP assays lay within sequences which were differentially expressed following inoculation with the fungal pathogen *Fusarium oxysporum* f.sp. *melongenae* [15], [16], with the genotyping effected via the high resolution melting (HRM) technique [17] and ran in a Rotor-Gene 6000 (Corbett Research, Mortlake, NSW, Australia) PCR machine. Of the microsatellite markers, 29 were taken from Nunome et al. [6], [7], 12 from Vilanova et al. [18] and one each from Stagel et al [19] and Frary et al. [20]. Microsatellite amplicons were separated on a LI-COR Gene ReadIR 4200 device, as described by Barchi et al. [10]. The three CAPS markers were all tightly linked to the *Rfo-sa1* gene, which confers resistance to *F. oxysporum* f.sp. *melongenae* [14]; the amplicons were separated on an AdvanCE™ FS96 capillary electrophoresis system (Advanced Analytical Technologies). The tomato RFLP loci [3] were assayed according to Bernatzky and Tanksley [21], while the COSII markers were developed by Wu et al. [22].

Differences between observed and expected segregation ratios were assessed using a χ^2^ test. Only markers associated with a χ^2^ value ≤ χ^2^_α = 0.1_, or slightly deviating from expectation (χ^2^_α = 0.1_< χ^2^ ≤ χ^2^_α = 0.01_) were considered, provided that their inclusion did not alter the local marker order. Loci suffering from significant segregation distortion (χ^2^ value > χ^2^_α = 0.01_), and any for which 30 or more of the 156 progeny were not successfully genotyped were excluded. JoinMap v4.0 software [23] was used to construct the map, based on a LOD threshold of 4.0. To determine marker order within a linkage group (LG), the JoinMap parameters were set at Rec  = 0.40, LOD  = 1.0 and Jump  = 5. Map distances were converted to centiMorgans (cM) using the Kosambi mapping function [24]. According to the known map locations of the RFLP, microsatellite and COSII markers, LGs were assigned to chromosome, and named E01 to E12. The quality of the map was first checked by using the nearest neighbour stress parameter in Joinmap, then by manual inspection to minimize the number of double recombination events, and finally by estimating pairwise recombination fractions using the R/QTL software package [25].

A blastN search of SNP, RFLP and COSII markers was made against the SL2.40 genome build published by the International Tomato Annotation Group (http://solgenomics.net/); for the RAD-derived SNPs, a 0.6 ratio between the number of identities and the query length was used as a cut off. A blastX search was carried out against the NCBI protein database, adopting a threshold of e^–15^. Finally, a blastN search was conducted of all the markers represented on the Fukuoka et al. [8] map to identify any in common between the two maps.

### Phenotypic traits evaluation, data and QTL analyses

The mapping population, along with both of the parental lines and their F_1_ hybrid, were field grown both in northern and southern Italian locations [Montanaso Lombardo (ML: 45 20′N, 9 26′E) and Monsampolo del Tronto (MT: 42 53′N; 13 47′E)] in 2009. In both trials, the material was arranged as a set of two randomized complete blocks with four replicate plants per entry per block (for the F_2_ individuals, replicates were achieved by establishing vegetative cuttings). The traits assayed were: adaxial leaf lamina anthocyanin (adlan), stem anthocyanin (stean), abaxial leaf lamina anthocyanin (ablan), calyx anthocyanin (calan), corolla colour (corcol), leaf venation anthocyanin (lvean) and fruit peduncle anthocyanin (pedan). Six of the seven traits were scaled from 0 to 3, while corcol was scored on a 1–5 scale, with “1” representing pink, “2” dark pink, “3” light violet, “4” violet-pink and “5” violet. Statistical analyses were performed using R software [26]. A conventional analysis of variance was applied to estimate genotypic/environmental effects based on the linear model *Y_i_*_j_  =  *μ* + *g*_i_ + *b*_j_ + *e*_ij_, where *μ*, *g*, *b* and *e* represented, respectively, the overall mean, the genotypic effect, the block effect and the error. Based on the F_2,_ the broad-sense heritability (*h^2^_BS_*) values were calculated as σ^2^_G_/( σ^2^_G_ + σ^2^_E_/n), where σ^2^_G_ represented the variance in *g* and σ^2^_E_ the residual variance and n the number of blocks. Correlations between traits were estimated using the Spearman coefficient, and normality, kurtosis and skewness assessed with the Shapiro-Wilks test (α = 0.05). Segregation was considered as transgressive where at least one F_2_ individual recorded a trait value higher or lower by at least two standard deviations than, respectively, the higher or lower scoring parental line.

Both interval [27] and MQM [28]–[30] mapping, as implemented in MapQTL v5 [31],were used for QTL detection. Putative QTLs were first identified using interval mapping, after which one linked marker per putative QTL was treated as a co-factor to represent genetic background control in the approximate multiple QTL model. Co-factor selection and MQM analysis were repeated until no new QTL could be identified. LOD thresholds for declaring a QTL to be significant at the 5% genome-wide probability level were established empirically by applying 1,000 permutations per trait [32]. Additive and dominance genetic effects, as well as the proportion of the variance explained by each QTL (PVE) were obtained from the final multiple QTL model. MapChart v2.1 software [33] was used to produce visualization of chromosomes and QTLs.

## Results

### Linkage analysis

**Figure 1:**
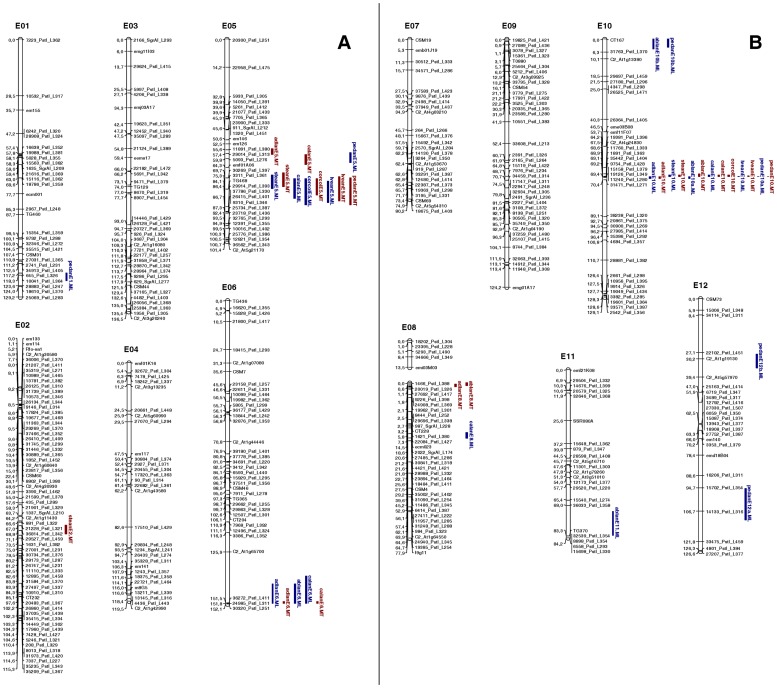
Linkage map of eggplant and graphical representation of the QTLs detected. A) chromosomes E01 to E06; B) chromosomes E07 to E12. Marker names are shown to the *right* of each chromosome, with map distances (in cM) shown on the *left*. Map positions of the QTL identified on each chromosome (or LG) are also given on the *right*. The length of the vertical bars represents the confidence interval of the QTL (LODmax-1 interval). QTL shown in *blue* were detected only at ML, and those in *red* only at MT.

Of the 384 SNPs included in the GoldenGate assay, 343 produced non-ambiguous data; the two replicated individuals included as internal controls produced completely consistent allele calls. The frequency of missed calls was on average 0.2%, with an extreme frequency of 5% occurring in one of the F_2_ segregants. Segregation was skewed (χ^2^ > χ^2^_α = 0.01_) for only 13 markers (seven SNPs, four microsatellites, one RFLP and one COSII), but in no case was the distortion enough (χ^2^ > χ^2^_α = 0.01_) to discard the data. The genotype data relating to the 431 informative markers were assembled into 12 major and one minor (5 markers) LG, comprising 415 loci (Table 1 and Fig. 1). The remaining 16 loci were associated as triplets (four SNPs and five microsatellites) or were unlinked; five of the microsatellites and two of the HRM markers were assigned to an LG but not ordered in the map. The location of the RFLP, microsatellite and COSII loci established from prior maps [3]–[7] allowed each LG to be assigned to one of the 12 eggplant chromosomes; the chromosome E08 map was formed by an unmerged major and minor LG (Fig. 1). The overall length of the map was 1,390 cM, with individual chromosomes ranging in length between 80.2 cM (E07) and 136.5 cM (E03); the number of loci per chromosome was highest in E02 (66) and lowest in E11 (22) (Table 1). The genome-wide mean inter-locus separation (discounting completely co-segregating ones) was 3.8 cM, varying from 2.0 cM (E02) to 7.0 cM (E12). An inspection of the pairwise recombination fractions revealed a well defined diagonal, implying that adjacent loci had the highest LOD; this confirmed the robustness of the linkage map.

**Table 1:** Parameters associated with the framework eggplant genetic map.

Chromosome	Length (in cM)	Number of markers	Average density (cM)	Gaps (>10 cM)	Gaps (>15 cM)	RAD tag SNP	HRM	SSR	CAPs	RFLP	COSII
E01	129.2	29	4.61	2	1	25		3		1	
E02	115.3	66	2.02	2	1	55		3	3	1	4
E03	136.5	37	4.01	1	1	30		4		1	2
E04	119.5	30	4.27	2	2	22	1	3			4
E05	101.4	32	3.76	1	1	27		3		1	1
E06	152.1	37	4.35	2	1	29		2		3	3
E07	80.2	25	3.64	2	0	19		3			3
E08	91.4	38	2.69	2	0	32	1	3		1	1
E09	124.2	39	3.65	2	0	34		2		1	2
E10	129.1	35	4.45	1	3	30		2		1	2
E11	84.3	22	4.68	2	1	16		2		1	3
E12	126.6	25	7.03	3	2	20		3			2
Average	115.8	34.6	3.82								
Total	1389.7	415				339	2	33	3	11	27

A blastN search against the tomato genome revealed that 223 of the 339 SNP loci (listed in Table S1) satisfied the cut off criterion applied, and thus, together with the RFLP and COSII markers (for a total of 261 loci), were suitable for assessing synteny and collinearity between the eggplant and tomato genomes. All major established syntenic relationships between the two genomes [4], [8] were confirmed (Fig. S1). Only 15 markers mapped to non-homologous tomato chromosomes (Table 2). When the RAD-derived SNP containing sequences were subjected to a blastN search of the Fukuoka et al. [8] loci, only two (31763_PstI_L370 and 18675_PstI_L403) could be associated (the former with SOL1236, mapping to chromosome E10 and the latter with est_cal05h22 on E07) (Fig. S2).

**Table 2:** Loss of synteny between eggplant and tomato.

Eggplant chromosome	Marker name	Tomato chromosome
E01	28908_PstI_L324	T04
E02	11363_PstI_L344	T00
E04	32672_PstI_L304	T07
	30804_PstI_L374	T08
E06	C2_At1g44446	T11
E09	3525_PstI_L303	T07
	23589_PstI_L280	T06
	33608_PstI_L213	T03
	7976_PstI_L234	T02
	34459_PstI_L314	T07
E10	11240_PstI_L280	T00
E12	6719_PstI_L347	T10
	18908_PstI_L337	T02
	15702_PstI_L354	T04
	27752_PstI_L387	T09

The table lists the15 loci which mapped to non-homogous locations in eggplant and tomato.

### Phenotypic variation and inter-trait correlations

A summary of the phenotypic performance and the derived *h^2^_BS_* values are listed in Table 3. The parental lines contrasted for each of the traits, as expected. ′305E40′ produced less anthocyanin than ‘67/3’ in its leaves and stems, and its corolla was pink to dark pink (′67/3′s was violet). The F1 hybrid's phenotype was intermediate between the two parents, except with respect to lvean, for which it more resembled ‘67/3’ in both MT and ML. Transgression among the F_2_ progeny was noted for pedan (six plants) in ML, and for adlan (six plants), stean (two plants) and lvean (two plants) in MT. In each case the transgression involved a lower level of pigmentation than in ‘305E40’. *h^2^_BS_* was overall high, ranging from 0.71 (lveanML) to 0.99 (pedanML) (Table 3). Significant inter-trait correlations were detected both within and across locations (Table 4). The least well correlated traits were adlan and corcol in MT (r^2^  = +0.23), and the most highly correlated were stean and calan in ML, and stean and lvean in MT (+0.86). The performance with respect to each trait was highly correlated between the two locations.

**Table 3:** Trait means, standard deviations (SD), coefficients of variation (cv) and broad sense heritability for the traits.

Trait	code	Environment	Parents means±SD		Significant mean difference among parental values (wilcoxon test)	F_1_	F_2_ population mean ± SD	cv	Skewness	SE	Kurtosis	SE	Heritability
			305E40	67/3									
Adaxial leaf lamina anthocyanin	adlan	ML	0±0	3±0	Yes: p<0.05	1.5±0	1.57±0.92	0.58	0.02	0.20	−1.26	0.39	0.93
		MT	0.5±0	3±0	Yes: p<0.05	1.5±0	1.5±0.83	0.55	0.23	0.19	−1.19	0.39	0.92
Stem anthocyanin	stean	ML	1.25±0.29	3±0	Yes: p<0.05	2.2±0.3	2.4±0.52	0.22	−0.54	0.20	−0.78	0.39	0.89
		MT	1±0	3±0	Yes: p<0.05	2.25±0.35	2.32±0.52	0.22	−0.78	0.19	0.20	0.39	0.82
Abaxial leaf lamina anthocyanin	ablan	ML	0.37±0.25	2.75±0.29	Yes: p<0.05	1.75±0.2	1.37±0.7	0.51	0.23	0.20	−1.10	0.39	0.85
		MT	0.5±0	3±0	Yes: p<0.05	2±0	1.5±0.61	0.41	0.01	0.19	−0.90	0.39	0.82
Calyx anthocyanin	calan	ML	1.12±0.25	3±0	Yes: p<0.05	1.8±3	2.2±0.55	0.25	−0.43	0.20	−0.71	0.39	0.90
		MT	0.75±0.35	3±0	Yes: p<0.05	1.75±0.35	2.02±0.68	0.34	−0.63	0.19	−0.64	0.39	0.88
Corolla colour	corcol	ML	1±0	5±0	Yes: p<0.05	3.9±0.15	3.95±1.61	0.41	−1.12	0.20	−0.58	0.39	0.94
		MT	1±0	5±0	Yes: p<0.05	4±0	3.57±1.6	0.45	−0.69	0.19	−1.12	0.39	0.84
Venation anthocyanin	lvean	ML	0.5±0.2	3±0	Yes: p<0.05	3±0.5	2.02±0.75	0.37	−0.52	0.20	−0.92	0.39	0.93
		MT	0.5±0	2.5±0.7	Yes: p<0.05	2.5±0.71	1.9±0.77	0.41	−0.52	0.19	−0.99	0.39	0.71
Fruit peduncle anthocyanin	pedan	ML	0.5±0	3±0	Yes: p<0.05	2±0	2.05±0.9	0.44	−0.69	0.19	−1.03	0.39	0.99
		MT	0±0	3±0	Yes: p<0.05	2.5±0.5	1.61±0.91	0.57	−0.30	0.19	−1.29	0.39	0.93

Skewness and kurtosis (with their standard errors (SE)) are also listed.

**Table 4:** Inter-trait Spearman correlations assessed in the mapping population.

Trait	Environment	Adlan	Stean	Ablan	Calan	Corcol	Lvean	Pedan
Adlan	ML	0,92	0,78	0,83	0,83	0,35	0,80	0,79
	MT		0,71	0,83	0,74	0,23	0,79	0,76
Stean	ML		0,80	0,67	0,86	0,45	0,84	0,76
	MT			0,70	0,78	0,43	0,86	0,74
Ablan	ML			0,83	0,75	0,26	0,69	0,69
	MT				0,77	0,33	0,78	0,71
Calan	ML				0,83	0,41	0,85	0,78
	MT					0,34	0,85	0,74
Corcol	ML					0,68	0,40	0,32
	MT						0,41	0,42
Lvean	ML						0,89	0,83
	MT							0,84
Pedan	ML							0,87
	MT							

The values in diagonal represent correlations of the same trait between the two environments. All correlations were significant (p<0.05).

### QTL detection and candidate gene analysis

Separate QTL analyses were performed for each location, resulting in the detection of 21 QTLs in ML and 18 in MT distributed over eight chromosomes (Table 5). QTL clusters were apparent on chromosomes E05, E06 and E10 (Fig. 1). Chromosome E05 harboured coincident major QTLs responsible for the expression of stean, lvean and corcol in both locations, while coincident major QTLs for all traits (except for corcol in ML) mapped to a site on chromosome E10. Some minor and location-specific QTLs were also found. Between one and six QTLs per trait was detected. Although the PVE ranged from 1.5% (pedanE01.ML) to 77.2% (lveanE10.MT), at least one major QTL (PVE values >10% and a LOD score >20) and one minor QTL could be identified for each trait – with the sole exception of corcol in ML, for which only one QTL could be mapped. The largest single QTL effect was associated with pedanE10a.ML (76.4% of the PVE). With the exceptions of steanE02.MT, pedanE12 b.ML and pedanE01.ML, all the positive alleles (increased anthocyanin content) derived from ′67/3′. The additive effects of all the QTLs were significant at p<0.05.

**Table 5:** QTLs detected in the mapping population.

Trait code	Montanaso Lombardo (ML)										Monsampolo del Tronto (MT)									
	GW	QTL	Chrom	Position (cM)	Locus	LOD	CI	PVE	A	D	GW	QTL	Chrom	Position (cM)	Locus	LOD	CI	PVE	A	D
Adlan	3.9										3.9	adlanE05.MT	5	57.383	29014_PstI_L313	5.94	55.5–59.4	4.2	−0.182	0.281
		adlanE06.ML	6	151.48	36272_PstI_L411	7.93	142−152	8.00	−0.282	0.287		adlanE06.MT	6	152.125	30320_PstI_L251	5.66	151–152	4	−0.226	−0.011
												adlanE08.MT	8	1.144	27692_PstI_L417	5.44	0.5–2	3.8	−0.176	0.246
		adlanE10.ML	10	69.39	15158_PstI_L379	36.98	69.1–69.9	60.60	−0.948	0.060		adlanE10.MT	10	69.39	15158_PstI_L379	44.96	69.1–69.9	60.9	−0.865	−0.049
Stean	3.9										3.9	steanE02.MT	2	71.05	29527_PstI_L459	4.92	68.7–73	4.40	0.151	−0.015
		steanE05.ML	5	69.73	30269_PstI_L397	14.59	68–75	14.80	−0.251	0.234		steanE05.MT	5	69.73	30269_PstI_L397	14.95	67–74	15.70	−0.272	0.200
		steanE10.ML	10	68.92	1891_PstI_L363	36.60	68.1–69	53.60	−0.487	0.252		steanE10.MT	10	68.92	1891_PstI_L363	32.50	68.1–69	45.60	−0.447	0.259
Ablan	3.8	ablanE06.ML	6	151.482	36272_PstI_L411	8.21	142–152	8.70	−0.209	0.259	3.8									
												ablanE08.MT	8	1.14	27692_PstI_L417	4.26	0–1.5	6.30	−0.216	0.097
		ablanE10a.ML	10	68.92	15158_PstI_L379	29.89	69.1–69.9	45.20	−0.642	−0.004		ablanE10.MT	10	68.58	11760_PstI_L333	23.1	67.5–69.3	46.0	−0.552	0.071
		ablanE10b.ML	10	0	CT167	6.59	0–5	6.80	−0.255	−0.101										
		ablanE11.ML	11	83.275	TG370	4.51	71–83	4.50	−0.177	0.227										
Calan	3.8	calanE05.ML	5	75.30	3311_PstI_L361	12.39	70–80	8.80	−0.213	0.147	3.8	calanE05.MT	5	59.81	5093_PstI_L276	5.32	58–63	3.4	−0.174	0.095
		calanE06.ML	6	151.48	36272_PstI_L411	4.48	138–151.7	2.70	−0.094	0.117		calanE06.MT	6	152.13	30320_PstI_L251	4.15	151–152	2.6	−0.131	0.097
		calanE08.ML	8	27.53	CSM4	5.11	25–28	3.40	−0.112	0.130										
		calanE10.ML	10	68.92	1891_PstI_L363	47.53	68–69	61.00	−0.553	0.290		calanE10.MT	10	68.92	1891_PstI_L363	52.25	68–69	74.1	−0.736	0.430
Corcol	4.5	corcolE05.ML	5	75.30	3311_PstI_L361	34.08	70–83	63.70	−1.545	1.490	4.0	corcolE05.MT	5	75.30	3311_PstI_L361	32.67	70–78	57.30	−1.539	1.227
												corcolE10.MT	10	69.13	35442_PstI_L404	4.08	68–69	4.50	−0.424	0.253
Lvean	3.7	lveanE05.ML	5	75.30	3311_PstI_L361	13.71	71–82	7.80	−0.274	0.189	3.9	lveanE05.MT	5	75.30	3311_PstI_L361	10.01	69–81	5.10	−0.226	0.162
		lveanE10.ML	10	69.13	35442_PstI_L404	58.94	68.6–69.1	73.90	−0.833	0.398		lveanE10.MT	10	68.92	1891_PstI_L363	61.80	68.5–69	77.20	−0.869	0.445
Pedan	4.2	pedanE01.ML	1	118.30	10041_PstI_L364	4.69	117–121	1.50	0.067	−0.191	3.8									
		pedanE05.ML	5	59.81	5093_PstI_L276	5.95	57–62	1.90	−0.110	0.227		pedanE05.MT	5	75.30	3311_PstI_L361	5.57	69.7–83	4.00	−0.221	0.222
		pedanE10a.ML	10	69.13	35442_PstI_L404	73.20	69.1–69.2	76.40	−1.029	0.551		pedanE10.MT	10	69.13	35442_PstI_L404	48.32	68.9–69.1	70.70	−0.998	0.404
		pedanE10b.ML	10	0.00	CT167	6.33	0–4	2.00	−0.191	−0.039										
		pedanE12a.ML	12	106.73	14133_PstI_L316	7.12	95–111	2.30	−0.190	0.020										
		pedanE12b.ML	12	30.23	C2_At1g19130	5.73	28–35	1.80	0.181	0.032										

For each trait the genome-wide LOD thresholds (GW) at p = 0.05 (as determined from 1,000 permutations) is indicated. The closest mapping marker to each QTL and which parent contributed positively to the trait are indicated, along with the LOD value of the QTL, the confidence interval (CI), the percentage of variation explained (PVE) and the additive (A)/dominance (D) contribution.

**Figure 2:**
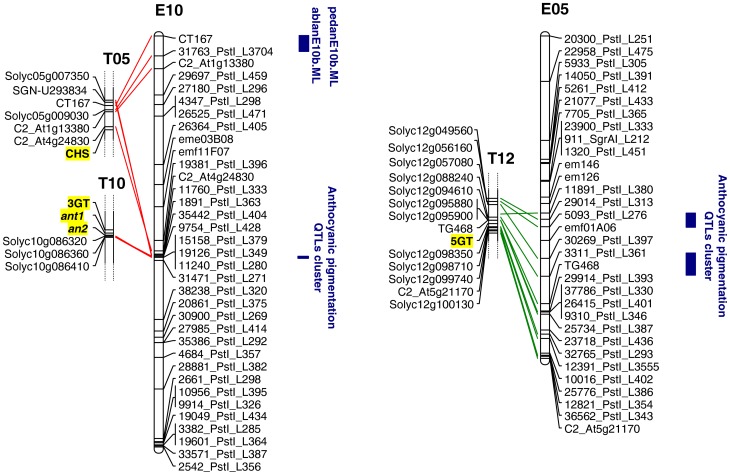
Synteny between eggplant chromosomes E5 and E10 and tomato chromosomes segments T5, T10 and T12. The physical location of the tomato genes encoding chalcone synthase (*CHS*), UDP-glucose anthocyanidin 3-0 glucosyltansferase (*3GT*), UDP-glucose anthocyanidin 5-0 glucosyltansferase (*5GT*) and the transcription factors *an2* and *ant1* is underlined. QTLs involved in anthocyanin pigmentation in eggplant are also shown. Distances on the eggplant chromosomes are given in cM, and on the tomato chromosome segments in Mbp.

A blastX search of the NCBI non-redundant protein database carried out for the seven marker loci on E10 and the two on E05 linked to the QTLs (Table 6), failed to highlight any known genes or transcription factors involved in anthocyanin synthesis (for a schematic view in tomato see Al-sane et al. [34]). A blastN search of the tomato genome sequence using the sequences of the loci mapping closest to the E10 ablan QTL (in MT) and adlan (both MT and ML) identified homologous sequences present on chromosome T10, while sequences mapping in the vicinity of the stean, calan and ablan QTLs detected in ML, and the lvean and pedan QTLs on chromosome E10 identified sequences present on chromosome T5 (Fig. 2). This confirmed the suggestion that chromosome E10 is a mosaic, composed of segments homologous to parts of chromosomes T5, T10 and T12 [4], [8]. Similarly, chromosome E05 appears to be a mosaic of chromosome T5 and T12 segments.

**Table 6:** BLAST results for marker sequences linked to an eggplant QTL.

Markers	Chromosome	BLAST result and annotation	p value
15158_PstI_L379	E10	NA	
11760_PstI_L333	E10	citrate synthase [*Nicotiana tabacum*]	7e-27
1891_PstI_L363	E10	NA	
35442_PstI_L404	E10	NA	
9754_PstI_L428	E10	Heat shock cognate 70 kDa protein 2	3e-101
19126_PstI_L349	E10	NA	
11240_PstI_L280	E10	cationic peroxidase 1 [*Vitis vinifera*]	2-e44
3311_PstI_L361	E05	hypothetical protein SORBIDRAFT_03g035660 [*Sorghum bicolor*]	1e-34
30269_PstI_L397	E05	glycosyltransferase [*Nicotiana tabacum*]	7e-60

To identify candidate genes underlying the eggplant E10 and E5 QTLs for anthocyanin accumulation identified in the present work, we investigate the available tomato sequence (*Lycopersicon esculentum* genome sequence build 2.40; http://solgenomics.net/organism/Solanum_lycopersicum/genome) in the tomato syntenic regions to search for genes and transcription factors known to be involved in anthocyanins pathways (Fig 2). Among the tomato genes / transcription factors involved in anthocyanin synthesis, the gene encoding chalcone synthase (CHS) gene is located on T5, distal to the region homologous to the chromosome E10 region harbouring QTLs for stean, calan, lvean and pedan (1891_PstI_L363 and 35442_PstI_L404). The gene encoding UDP glucose anthocyanidin 3-0 glucosyltansferase (3GT), *an2* and *ant1* are all located on chromosome T10, proximal to the E10 region containing 15158_PstI_L379 (linked to an ablan QTL in MT and an adlan QTL detected in both locations). With respect to corcol, Doganlar et al. [35] were able to identify a QTL on chromosome E05. Aligning the two maps showed that this QTL did not coincide with either corcolE05.ML or corcolE05.MT (data not shown). A blastN search using the sequences of the loci most closely linked to the chromosome E05 corcol QTL did however reveal a syntenic locus on tomato chromosome T12, in a region harbouring the gene encoding the anthocyanin synthesis-associated enzyme UDP glucose anthocyanidin 5-0 glucosyltansferase (GT) (Fig. 2).

## Discussion

### Genetic map construction

Eggplant remains a “genomic orphan species” having enjoyed very little investment to date in genome sequencing and molecular genetics. A robust linkage map represents the springboard for genomic investigation and targeted breeding. As in many crop species, the level of intraspecific polymorphism present in eggplant is low [19]. The same mapping population utilized here had been previously used to assign 348 markers (mostly AFLP loci) to 12 major LGs, but the map suffered from extensive marker clustering, particularly on the LG corresponding to chromosome E2 [10].Current DNA sequencing technologies have substantially lowered the cost of sequence acquisition, while the development of automated SNP platforms has revolutionized genotyping. The RAD tag approach has therefore allowed for the definition of a large number of SNP loci [12], a proportion of which were incorporated in the GoldenGate SNP array used here. As a result, the AFLP data have become redundant and they have been discarded for the development of the present map.

The mapping population proved to be largely free of segregation distortion, with only 4% of the markers showing evidence of skewing (and none needing to be discarded on account of severe distortion). The global genetic length defined by the map was 1,390 cM, a length not dissimilar to that obtained by both Wu et al. [4] and Fukuoka et al. [8], but considerably longer than the AFLP-based one we previously constructed [10]. The 13 LGs identified were assignable to the full complement of 12 chromosomes. Only E08 chromosome featured more than one LG; this assignment was made possible by the presence of two SSR markers, (i.e. emi03M03 and ecm023,) previously reported to be on the same chromosome by both Nunome et al. [7] and Fukuoka et al.[8]. The use of common (RFLP and COSII) markers allowed for the full alignment of the current map with the one developed by Wu et al. [4], while the position of microsatellite and COSII markers (and two SNP loci) helped achieve the alignment with the Fukuoka et al. [8] map for all but one of the chromosomes (E12) (Fig. S2). The microsatellite locus emd18B04 was assigned to E12, but is given as being on E09 by Fukuoka et al. [8]; this discrepancy could reflect genetic differences between the pairs of mapping parents, and/or may have arisen as a statistical artefact, reflecting a non-identical choice of mapping parameters.

The positions of the homologues of the SNP, RFLP and COSII loci in the tomato genome agreed well with previous analyses [4], [8]. The 15 markers which appeared to break synteny may reflect the outcome of transposon-mediated transposition, as has been recorded in both eggplant and pepper [4], [36].

### Mapping the QTLs underlying anthocyanin pigmentation

The genetic basis of anthocyanin synthesis and accumulation has been widely explored in the *Solanaceae* [37]–[43]. Anthocyanins are involved in floral pigmentation, protection against UV light induced injury [44]–[46], tolerance of low temperature, nutrient deficiency and defence against pathogen attack [47]–[49]. As part of the human diet, they are recognized as having anti-inflammatory and antioxidant properties [38]. The genetic control of the accumulation and distribution of anthocyanin in eggplant was long thought to be complex [50], [51], involving at least three major and five minor loci; epistatic interactions and/or pleiotropic effects have also been implicated. The advent of marker-based linkage maps has begun to clarify the situation, with the first report of a QTL influencing anthocyanin content having been provided by Doganlar et al. [35]. Based on the current map, it has been possible to identify a number of QTLs underlying anthocyanin accumulation in several tissues / organs. The cluster of QTLs assigned to chromosome E10 is likely the same as that proposed by Doganlar et al. [35]. The location of the phenotyping trials did not greatly influence the outcomes (in other words, there was only a minor component of GxE variation present), with the performance of the parents and their F_1_ being very similar in each of the two environments. The high *h^2^_BS_* value associated with all the traits along with the correlated trait performances between the two sites can be taken as evidence that soil and climate variation – to the extent that the two sites differed for these – had little influence on the phenotypic outcome. The *h^2^_BS_* values were higher than 80% for all the traits; only for lvean it was markedly higher in ML (0.93) than in MT (0.71) as in the latter location an higher phenotypic variation was detected. Heritability values for some of the traits in study have been recently reported [52].

Transgression was rare, and always in the direction of the less pigmented parent (305E40). As previously reported [53], transgressive genotypes outcome from the combination of alleles from both parents that have effect on the same direction. The effect of such allele combinations was tested regarding the graphical genotypes at the detected QTLs of transgressive individuals. The two transgressive individuals with respect to stean and lvean QTLs in MT carried marker alleles inherited from different parents, both acting to decreasing the expression of the trait. However, the transgressive individuals for adlan at MT and pedan at ML were not found to completely pyramide known alleles responsible to increase the traits, implying that some minor QTLs still remain to be identified.

Collard et al. [54] have suggested that a QTL should only be classified as “major” if it can account for >10% of the PVE. A more nuanced definition of “major” requires that the QTL can be shown to be stable across multiple seasons/locations [55]–[57]. Among the 21 QTLs identified in ML and the 18 in MT, at least one major QTL per trait was identified. The LOD score associated with the least convincing of these was 23.3 (ablanE10.MT) and the most convincing was 73.2 (pedanE10a.ML); the PVE varied from ∼46% (steanE10.MT) to ∼77% (lveanE10.MT). Their key role and stability is respectively confirmed by the high PVE and from their localization in the same chromosome region in both locations. The stability of some of these QTLs is promising in terms of using them in the context of marker-assisted selection.

Anthocyanin pigmentation in eggplant, at least in the cross used for mapping, is controlled by loci on chromosomes E05, E06 and E10. The region between 68 and 70 cM of E10 (defined by seven marker loci) is particularly prominent for anthocyanin production and accumulation throughout the plant (except in the corolla). The major site for corcol was within a ∼13 cM region of chromosome E05 (defined by two marker loci) which harboured both major QTLs expressed in both locations and minor QTLs responsible for lvean (in both locations) and pedan (just in MT). The implication is that the corcol trait is controlled by one or more genes which are not directly responsible for pigmentation of the leaf, stem or calyx. Since both parental lines produce anthocyanin pigmented fruits, no detectable variation in fruit pigmentation was observed in the F2 progeny.

QTLs linked to related traits have a tendency to co-localize [35]. This situation arose on E10, where QTLs for six of the seven traits all mapped to the same region. The exception was corcol, where the largest QTL mapped to chromosome E05 in the vicinity of RFLP marker TG468. The minor QTLs detected were concentrated on chromosomes E05, E06, E08 and E10. Some were expressed in both locations (e.g. adlanE06, steanE05, calanE05, calanE06, lveanE05 and pedanE05), while others were location-specific (e.g. adlanE05.MT or steanE02.MT). Two pairs of such QTLs (calanE05.MT / calanE05.ML and pedanE05.MT / pedanE05.ML) mapped to the same chromosome in both locations, but not to a comparable intra-chromosomal position.

### Synteny and putative orthologous QTLs in other Solanaceae genomes

Most of the genetic analysis relating to anthocyanin pigmentation in the *Solanaceae* has been carried out to date in potato, pepper and tomato. In the former, van Eck et al. [42], [58] were able to identify four loci (*P*, *I*, *R* and *F* ) required for anthocyanin synthesis in the tuber's skin and flowers. In pepper, the gene A (which is a component of the anthocyanin synthesis pathway) was shown by Borovsky et al [37] to encode a MYB transcription factor homologous to petunia *an2*. In tomato, 13 genes related to anthocyanin synthesis have been described by De Jong et al. [41]. The gene encoding chalcone synthase has been mapped to T5 in the vicinity of the RFLP locus TG60 and several genes have been mapped to T10, underlining the extent of synteny shown by this genomic region across *Solanaceae* family. These data enabled both candidate orthologues for three of the above potato genes to be proposed, and for predictions to be made with regard to orthologue identity in tomato (*ag* locus [59]), pepper (*A* locus [39] ) and petunia, (*an2* [60]).

In eggplant, it was suggested many years ago that anthocyanin production, distribution and accumulation are primarily under the control of the genes *D*, *P* and *Y*, and that the intensity of pigmentation is further influenced by the genes *Ac*, *Dil1*, *Dil2*, *Puc* and *Sac* [50], [51]. The anthocyanin-related QTL clusters on chromosomes E06 and E10 were suggested by Doganlar et al. [35] to reflect pleiotropy rather than to the presence of independent loci. Here, evident synteny between the critical regions on E10 and E05 and their equivalent regions in tomato supports the use of the tomato genome sequence as a surrogate for eggplant. The recognition in these tomato genomic regions of genes and transcription factors involved in anthocyanin synthesis can be used to identify eggplant candidate genes for anthocyanin accumulation in the stem or leaf, or for the determination of corolla colour. Validation of these candidates will of course need precise mapping, followed by detailed expression studies.

## Conclusions

In spite of the low level of intraspecific allelic variation present in eggplant, the use of RAD tags has made it possible to generate a large set of informative SNP markers. The length of the newly developed map, and the inferred syntenic relationships between the eggplant and the tomato genomes were largely consistent with the conclusions drawn by Fukuoka et al. [8] and by Wu et al. [4]. A number of major QTLs have been located, which together explain the majority of the phenotypic variance shown by the mapping population for each trait. This exercise confirmed the reliability of our map for the future identification of the genetic bases of breeding traits for marker assisted selection programmes. Synteny with tomato should allow the ready identification of candidate orthologues for the chromosome E10 and E05 QTLs related to anthocyanin accumulation. The expanded genetic map should also be useful for further QTL and candidate gene discovery.

## Supporting Information

Figure S1 **Comparative maps and syntenic relationships between eggplant and tomato chromosomes.** Each eggplant chromosome (in white) and its corresponding tomato physical chromosome (in yellow) are connected by solid lines (in green). Distances on the eggplant chromosomes are given in cM, and on the tomato chromosome segments in Mbp.
(PDF)

Figure S2 **Eggplant maps alignment.** Alignment of the current genetic map (white chromosome, in the middle) with that constructed by Fukuoka et al [8] (in blue, on the right) and the ones from Wu et al. [4] (in yellow, on the left). Markers shared by maps are shown and their positions connected by a line.
(PDF)

Table S1 **Parameters associated to the 261 loci used for synteny and collinearity between the eggplant and tomato genomes.**
(XLSX)
