# Supplementary figures and images for: A RAD Tag Derived Marker Based Eggplant Linkage Map and the Location of QTLs Determining Anthocyanin Pigmentation
Source: PLoS One. 2012 Aug 17;7(8):e43740. doi: 10.1371/journal.pone.0043740 (PMC3422253; doi:10.1371/journal.pone.0043740)

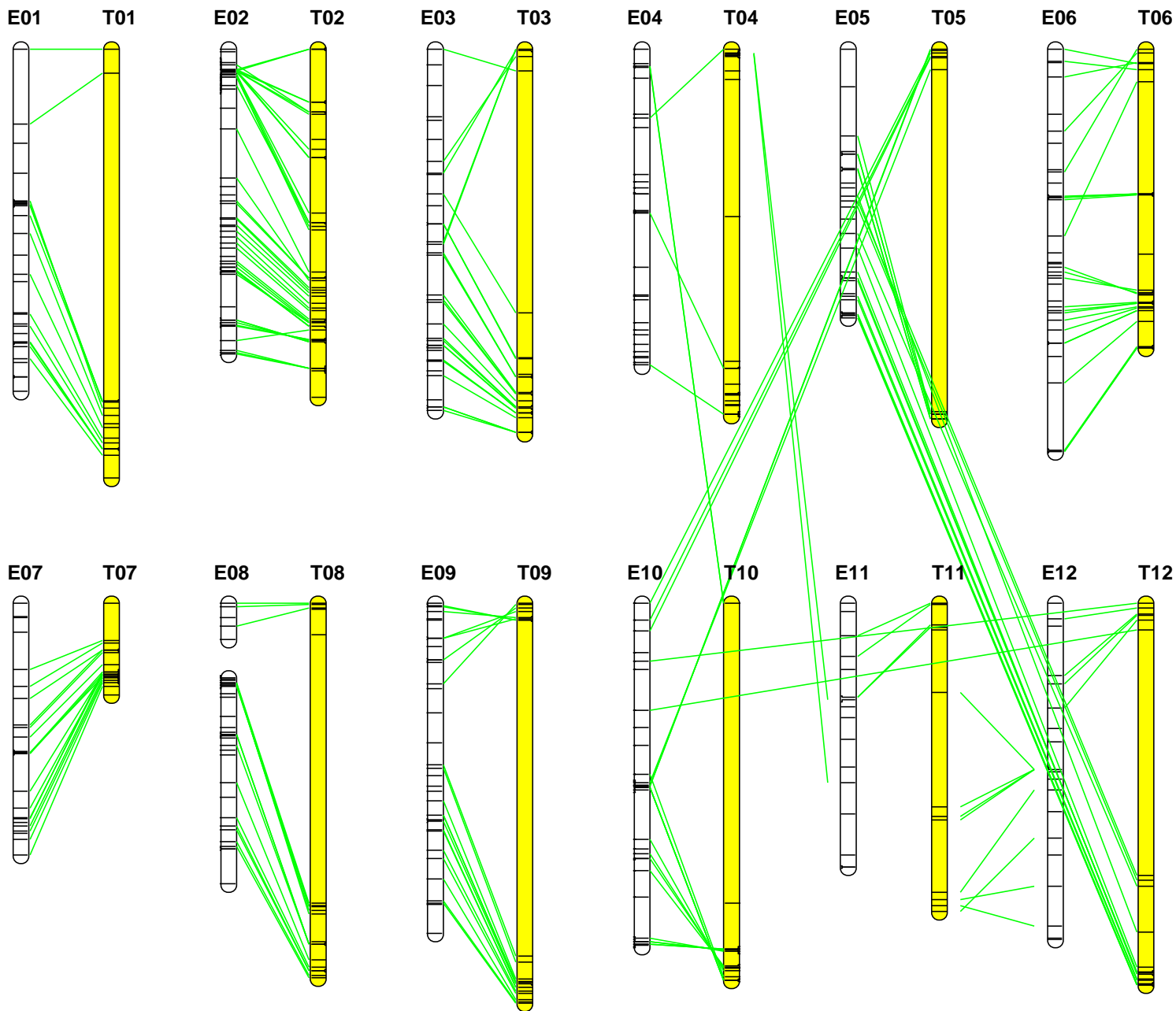

Supplement: Figure S1 — Comparative maps and syntenic relationships between eggplant and tomato chromosomes. Each eggplant chromosome (in white) and its corresponding tomato physical chromosome (in yellow) are connected by solid lines (in green). Distances on the eggplant chromosomes are given in cM, and on the tomato chromosome segments in Mbp. (PDF) [file pone.0043740.s001.pdf]

E01

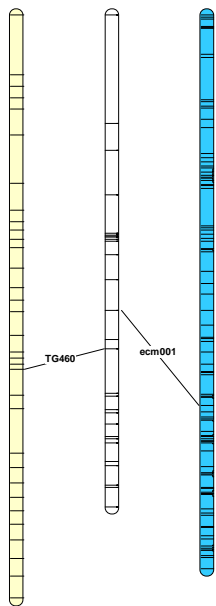

E02

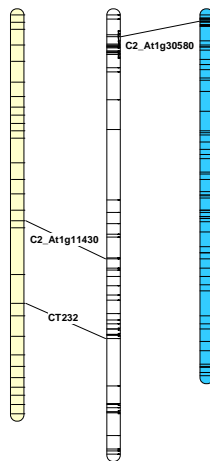

E03

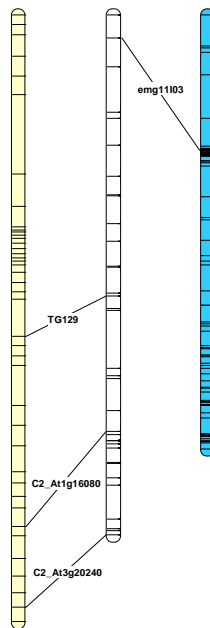

E04

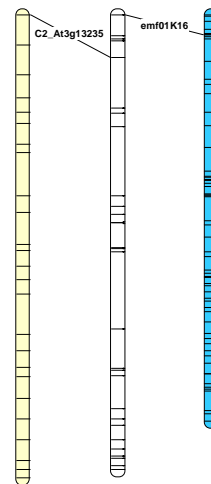

E05

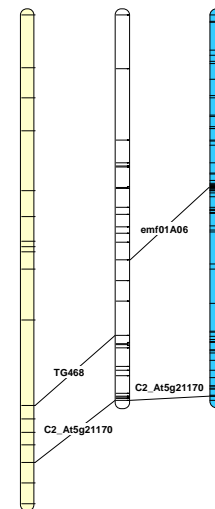

E06

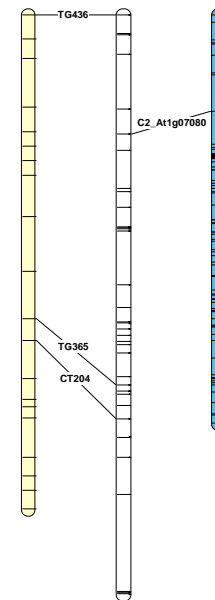

E07

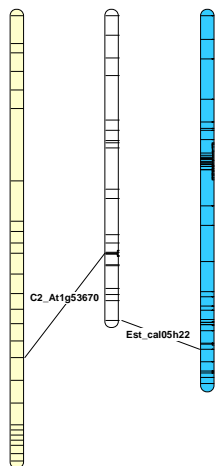

E08

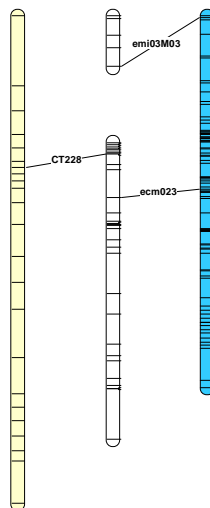

E09

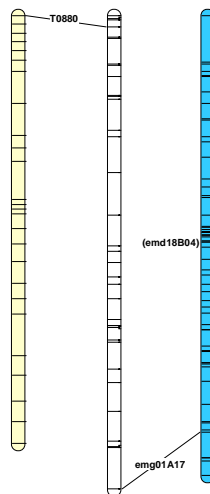

E10

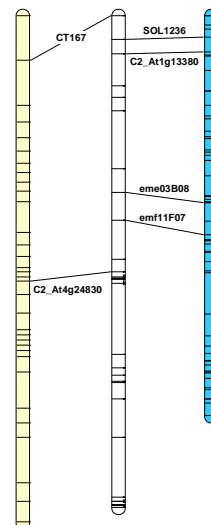

E11

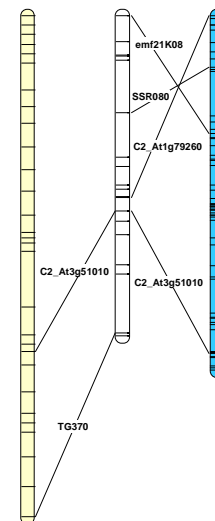

E12

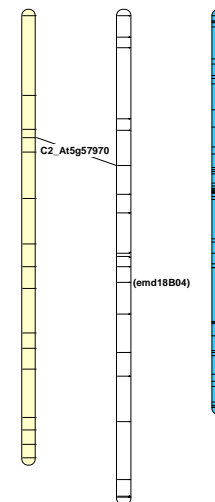

Supplement: Figure S2 — Eggplant maps alignment. Alignment of the current genetic map (white chromosome, in the middle) with that constructed by Fukuoka et al [8] (in blue, on the right) and the ones from Wu et al. [4] (in yellow, on the left). Markers shared by maps are shown and their positions connected by a line. (PDF) [file pone.0043740.s002.pdf]
